# Transitioning to Adult Services for Youth With Medical Complexity: A Practice Issue Viewed Through the Lens of Transitions Theory

**DOI:** 10.1177/08943184211010454

**Published:** 2021-07-02

**Authors:** Lin Li, Patricia H. Strachan

**Affiliations:** 1 PhD Student, School of Nursing, McMaster University, Hamilton, ON, Canada; 2 Associate Professor, School of Nursing, McMaster University, Hamilton, ON, Canada

**Keywords:** medical complexity, middle range theory, nursing theory, transition, youth

## Abstract

As youth with medical complexity transition to adult services, their extensive support networks are disrupted, leaving them vulnerable to care gaps. Within the setting of a pediatric complex care clinic in Ontario, Canada, the authors conducted a needs assessment guided by transitions theory to better understand the movement to adult services for youth and their families. The authors here describe the application of transitions theory and critique the theory’s usefulness for understanding the transition to adult services for youth and their families.

Advancement of nursing science is driven by inquiry and knowledge generation directed toward a unique disciplinary focus. Nursing theories can be defined as sets of related ideas focused on the reality of nursing (Smith & Liehr, 2014). While grand theories grapple with defining nursing’s disciplinary focus, middle-range theories are concerned with more concrete concepts (uncertainty, self-efficacy, and symptom management) (Smith, 2014). By focusing on a specific dimension of nursing, middle-range theories are more practical for providing structure, direction, and rationale for specific nursing practice and research issues. Thus, middle-range theory has an important place in the generation of substantive disciplinary knowledge and the linking of theory to practice.

Ongoing development of nursing theory and theory-guided practice relies on critical discussion and application of nursing theory to both research and practice situations. However, there are very few published examples of nursing practice viewed through the lens of a middle-range theory (Liehr & Smith, 2017). In this article, the authors describe and critique the application of a middle-range theory to a practice issue. Specifically, the authors evaluate the adequacy and usefulness of the transitions theory (TT) (Meleis et al., 2000) to guide a needs assessment aimed at understanding the transition to adult services for youth and families attending a pediatric complex care clinic.

## The Practice Issue

Children and youth with medical complexity (YMC) are individuals with complex chronic health conditions who have substantial family-identified service needs, functional limitations, and high health resource utilization (Cohen et al., 2011). They typically require extensive caregiver support and high-intensity medical management in order to remain healthy. An example of a child with medical complexity may be one who has a rare genetic disorder affecting multiple organ systems, is developmentally delayed, and has cognitive and physical limitations. These children and youth are usually dependent on medical technologies for vital bodily functions: For example, they may require a tracheostomy or ventilator for breathing, a gastrostomy tube and pump for feeding, and wheelchairs and mechanical lifts for mobility. Furthermore, although they make up less than 1% of all children and youth, they disproportionately account for one-third of all pediatric healthcare costs in Ontario (Cohen et al., 2012).

Caring for these children and youth frequently requires numerous services and providers across multiple care settings and sectors (health, social, and education sectors). Within Ontario, the Provincial Council for Maternal and Child Health (2015) has implemented the Complex Care for Kids Ontario (CCKO) strategy, which has established interdisciplinary pediatric complex care teams to provide comprehensive, coordinated care for these children, youth, and their families. However, due to the lack of equivalent adult complex care services, when YMC age out of the pediatric health system, this comprehensive care management does not follow them into adulthood. Furthermore, many of their other providers and services also change, disrupting their extensive support networks and leaving them vulnerable to care gaps. For the increasing population of YMC, the transition to adult services is both an urgent practice issue and an opportunity for quality improvement in health systems. As a first step in addressing this issue locally, we conducted a needs assessment within a complex care clinic at a tertiary pediatric health center in Ontario, Canada.

TT was used as a guiding theoretical framework to structure the needs assessment. The authors selected TT for its conceptual fit with the practice issue and its relevance to nursing. There are four major domains of the theory (nature of transitions, transition conditions, patterns of response, and nursing therapeutics) that relate to this particular transition. The authors decided that an examination of the transition to adult services through the lens of TT would provide insight into what a typical transition looks like (*nature of transitions*), what makes the process easier or more difficult (*transition conditions*), how a healthy transition is defined (*patterns of response*), and how the experience can be improved (*nursing therapeutics*). The purpose of this article is to describe and critique the application of TT to the transition to adult services for YMC and their families.

## Overview of Transitions Theory

### Background and Assumptions

Development of TT began in the 1970s with Meleis’ theoretical work on role supplementation and research on immigrant health (Im, 2014). Chick and Meleis (1986) defined transition as “a passage from one life phase, condition, or status to another” (p. 239). Subsequently, Schumacher and Meleis (1994) developed a transitions framework, which was further refined into a middle-range theory by drawing on the results of five nursing studies exploring diverse transitions (Meleis et al., 2000). The purpose of this middle-range theory is “to describe, explain, and predict human beings’ experiences in various types of transitions” (Im, 2014, p. 254). TT is grounded in ideas from role theory, lived experience, and feminist postcolonialism (Meleis, 2015). Role theory provides a basis for understanding how people transition from one role to another; lived experience situates TT within an epistemological paradigm that values subjective, experiential knowledge; and feminist postcolonialism considers how the intersection of race, class, and gender influence how people experience transitions (Meleis, 2015).

TT is underpinned by several key assumptions. Drawing from symbolic interactionism, the theory is based on the assumption that an individual’s response to a transition is influenced by interactions with others (Meleis, 2015). Transitions are both an effect of change and a cause for changes in roles, identities, situations, behaviors, and relationships (Im, 2014). Additionally, all transitions involve a temporal process that begins at or before a trigger event and has a fluid end point (Im, 2014; Meleis, 2015). Lastly, TT assumes that individuals are capable of learning new skills and adapting to new roles (Meleis, 2015).

### Concepts and Propositions

The six major concepts defined in TT include types and patterns of transitions, properties of transition experiences, transition conditions, process indicators, outcome indicators, and nursing therapeutics (Meleis et al., 2000). These concepts are grouped into four domains, and their relationships are illustrated in a model (see Figure). *Types of transitions* that nurses encounter can be developmental, situational, health/illness-related, or organizational. Nurses also need to consider *patterns of transitions*, which can be described as multiple, simultaneous, sequential, and/or related. While each transition is unique, properties that are essential to all transitions include awareness, engagement, change and difference, transition time span, and critical points and events (Meleis et al., 2000). These properties are interrelated, and changes in one property may affect another (engagement relies on awareness, and critical events may define transition time span) (Im, 2014). By inquiring into the *nature of transitions*, we hoped to gain insight into what a typical transition looks like for YMC and their families.

**Figure.**
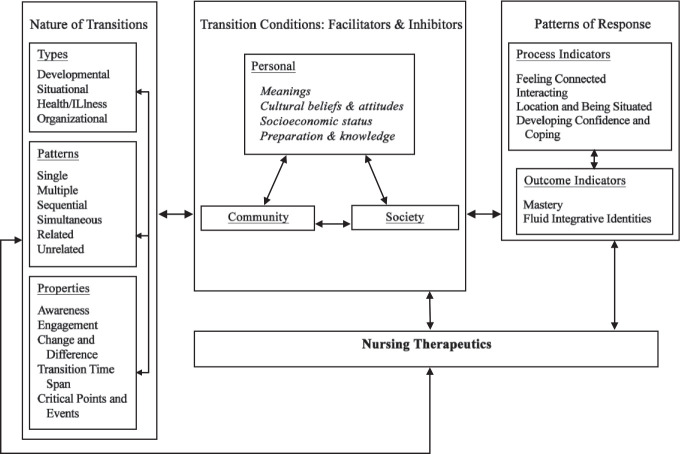
Transitions: A Middle-Range Theory. Reprinted from “Experiencing Transitions: An Emerging Middle-Range Theory,” by A. I. Meleis, Sawyer, L. M., Im, E. O., Hilfinger Messias, D. K., & Schumacher, K., 2000, *Advances in Nursing Science*, *23*(1), pp. 12-28. Reprinted with permission.

*Transition conditions*, or facilitators and inhibitors, are personal, community, and societal factors that mediate an individual’s response to a transition. Personal conditions include meanings attributed to the transition, cultural beliefs and attitudes, socioeconomic status, and preparation and knowledge related to the transition (Meleis et al., 2000). Examples of community conditions are community resources, support from others, and access to role models (Meleis, 2015; Meleis et al., 2000). Societal conditions refer to social norms, including stigma and stereotypes attached to the transition (Meleis et al., 2000). The authors examined *transition conditions* to discover what makes the transition to adult services easier or more difficult.

*Process indicators* include feeling connected, interacting, location and being situated, and developing confidence and coping (Meleis et al., 2000). *Outcome indicators* are defined as mastery of new skills and the emergence of a fluid integrative identity resulting from identity reformulation during the transition process (Meleis et al., 2000). Combined, process, and outcome indicators describe *patterns of response* that characterize a healthy transition. These responses are shaped by the *nature of transitions*, *transition conditions*, and *nursing therapeutics. Patterns of response* helped to define what a healthy transition means for YMC, their families, and their providers.

Lastly, although the concept of *nursing therapeutics* is not fully developed in the original middle-range theory, Meleis (2015) has since created an intervention framework to address this important gap. In this framework, nursing interventions can be preventative or therapeutic and include the following strategies: clarifying roles, competencies, and meanings; identifying milestones; mobilizing support; and debriefing. In the TT model, nursing therapeutics are linked to all other concepts, implying that nurses can positively influence many aspects of transition by establishing effective reciprocal relationships with their clients (Im, 2014). The authors used the domain of *nursing therapeutics* to understand how current and potential interventions could support the transition to adult services.

## Application of Transitions Theory

A needs assessment was conducted to identify current practices and resources available that support transition as well as existing gaps and potential strategies to address these gaps. Three different activities informed this project: (a) interviews with local stakeholders, (b) a literature review, and (c) a survey of other institutions for benchmarking purposes. In consultation with the Hamilton Integrated Research Ethics Board, this project was deemed to be quality improvement and was exempt from ethics review. The authors only applied TT as a framework to guide the interviews with stakeholders; as such, only this component of the larger project is discussed.

### Transitions Theory as an Interview Guide

The purpose of interviewing stakeholders was to gain a better understanding of practices, resources, and gaps in the local context. We sought perspectives from those who work closely with YMC and their families either at the tertiary pediatric health center or in the surrounding communities. The primary author (L.L.) conducted 15 in-person or phone interviews, and one person chose to respond to the interview questions via email. A total of 19 individuals participated, representing both tertiary and community settings, health and social sectors, children’s and adult services, and family and provider perspectives. We utilized concepts from TT to formulate the interview questions (Table 1). The primary author (L.L.) took notes of the salient points discussed during the interviews and provided participants with a summary to check for accuracy. These notes were then examined for common themes and unique experiences, and the findings were related back to TT. Interview findings paired with concepts from TT are shown in Table 2.

**Table 1.** Interview Questions Developed from Transitions Theory.

Transitions Theory Concept(s)	Interview question(s)
Types and patterns of transition	Are youth required to transition out of your organization’s services upon reaching adulthood? What are your policies around transition?
Properties of transition experiences	At what age is the transition process initiated? (*transition time span*) Are you aware of what other organizations or services/sectors do to facilitate transition? (*awareness* of related transitions)
Transition conditions	What factors make the process easier or promote a successful transition? (*facilitators*) What factors make the process more challenging or hinder a successful transition? (inhibitors) Are there any major gaps or unmet needs that you can identify and what might be strategies to address these? (inhibitors, nursing therapeutics)
Process & outcome indicators	How do you know the transition was successful? How do you define a successful transition?
Nursing therapeutics	Do you have a standard process for transitioning youth from children’s to adult services? If not, do you have your own process? Who is responsible for coordinating transition? What are the components of the transition process?

**Table 2.** Interview Findings Interpreted Through the Lens of Transitions Theory.

Transitions Theory Concepts	Interview Findings
*Types and patterns of transitions*	
Types	Developmental and situational
Patterns	Multiple and simultaneous
*Properties of transition experiences*	
Engagement	Family engagement with transition planning is important
Change and difference	Changes occur in funding, providers, services, decision-making, roles, and relationships
Transition time span	Starting the process early or late influences transition experiences
Critical points and events	Initiation of transition planning and transfer event
*Transition conditions*	
Personal/family	Level of family engagement; starting the process early; being knowledgeable about the process and services; socioeconomic status
Community	Relationships that continue across the lifespan; collaborative partnerships; community resources (e.g., children’s centers and family support groups); urban/rural setting
Society	Waitlists (for psychoeducational assessment, funding, programs); stigma from providers; lack of support for adult providers
*Process indicators*	
Feeling connected	Families are supported and connected to agencies
Interacting	Families have access to services and funding
Developing confidence and coping	Families feel empowered
*Outcome indicators*	
Mastery	High quality of life, happiness, and well-being for the youth and family
Fluid integrative identities	Services supporting meaningful activities in adulthood (e.g., education, employment, or activity programs); youth and families moving forward with their lives in typical ways
*Nursing therapeutics*	
Clarifying roles, competencies, and meanings	Exploring the youth’s and family’s goals (e.g., for education, community participation, independent living)
Identifying milestones	Ensuring that applications and assessments are done on time
Mobilizing support	Assisting families to apply for funding and connect with adult providers and services
Debriefing	Following up with families after transfer

### Transitions Theory as an InterpretiveFramework

While using TT to interpret the interview results, one limitation that became apparent is the theory’s focus on the individual, rather than the family. Families of YMC are heavily involved in their care and devote tremendous amounts of time and effort to care coordination and providing direct care at home (Schall et al., 2020). Thus, the needs of YMC are closely interrelated with those of their families. To make TT more relevant to YMC and their families, we examined *personal and family* conditions, rather than only *personal* conditions.

#### Nature of Transitions

By learning about the policies and scope of services offered by different organizations, it became apparent that YMC experience *multiple* service transitions *simultaneously*. However, the starting and ending points of these transitions do not always align. We identified two common *critical points and events* in these service transitions, (a) initiation of transition planning and (b) the transfer event, which indicates that the youth and family are no longer receiving children’s services. The age at which these critical points occur varies between providers and organizations, and this affects the *transition time span*. There are also a number of major *changes* associated with the transition to adult services, including changes in funding, providers, amount and types of services, decision-making, and roles and relationships. Things that do not change are viewed positively. For example, family members expressed that they value long-standing relationships with providers that continue across the lifespan. Additionally, providers often reported that the level of family *engagement* affects how smoothly the transition progresses.

#### Transition Conditions

*Personal and family* conditions that facilitate healthy transitions are starting the process early and being knowledgeable about the transition process (*preparation and knowledge*). *Community* facilitators include collaborative partnerships and availability of resources such as children’s centers and family support groups. The most commonly cited inhibitors are time and money, and these are often interrelated. For example, there is a lengthy waitlist to receive a publicly funded psychoeducational assessment, which is a requirement for funding and services (*societal* inhibitor). However, if families have the financial means to do so, they may pay out of pocket to have this assessment done sooner (*socioeconomic status*). Another *community* inhibitor is living in a rural area, where there are fewer resources and specialists compared to urban centers. Lastly, stigma is a major *societal* inhibitor, as it was reported that many adult providers and day programs are unwilling, or do not have the capacity, to accept individuals with medical complexity. This could be partially due to the lack of resources and support for adult providers in caring for YMC. For example, while there is a multidisciplinary team to support care management of YMC in the pediatric system, this responsibility for primary care usually falls on a single practitioner once the youth ages out of the pediatric system.

#### Patterns of Response

Families and providers have similar definitions of a successful transition, and these encompass healthy *patterns of response* for both youth and their families. For example, transition is viewed as successful if youth are able to engage in meaningful activities (*fluid integrative identities*) and maintain a high quality of life (*mastery*). Family indicators include feeling empowered (*developing confidence and coping*), being supported and well-connected (*feeling connected*), and receiving funding in order to access services (*interacting*). Lastly, a successful transition results in both the youth and their family experiencing happiness and well-being (*mastery*) and moving forward with their lives in typical ways (*fluid integrative identities*).

#### Nursing Therapeutics

In her more recent work, Meleis described four nursing interventions that can be used to promote healthy transition processes (Meleis, 2015), three of which align with transition planning activities that were commonly described by providers. These activities include exploring the youth’s goals for community participation and education (*clarifying roles, competencies, and meanings*), ensuring that a psychoeducational assessment is done by age 16 (*identifying milestones*), and assisting families to identify adult providers and apply for funding (*mobilizing support*). The fourth intervention, *debriefing*, was not reported as often as the other three, and only two providers described actively following up with families as a standard practice after transfer. It is also important to note that although these strategies are defined as nursing interventions, it was found that many nonnurses are delivering these interventions.

## Utility of Transitions Theory

The authors critiqued the utility of applying TT to practice issues using Chinn and Kramer’s (2015) framework for critical reflection. Five fundamental questions guide the critical reflection, which collectively ask, “Is the theory *clear, simple, general, accessible*, and *important?*” These questions are easy to understand, and they form the basis of a practical assessment of a theory’s usefulness and relevance for nursing. Chinn and Kramer (2015) assert that the value of a theory should not be determined by normative criteria (standards that imply “good” or “bad” judgment) but rather by how well a theory works in relation to some purpose. Therefore, the focus of the critique was on the usefulness and relevance of TT for informing the needs assessment. Strengths and limitations of TT that were not captured in Chinn and Kramer’s framework are also described.

The *clarity* of a theory can be analyzed in terms of semantic or structural clarity and consistency. *Semantic clarity* and *consistency* refer to how clearly concepts are defined and whether the meanings of concepts are used consistently throughout the theory. *Structural clarity* and *consistency* can be determined by whether the concepts are linked to create a coherent whole and to what extent these relationships make logical sense (Chinn & Kramer, 2015). Overall, TT has high *semantic clarity* and *consistency*. The concepts were easy to understand and apply to the practice issue, and their meanings were used consistently across the theory and the interviews. For example, it is clear that the transition to adult services involves multiple situational transitions that occur simultaneously (transitions between healthcare providers, social services, and government funding). These transitions also coincide with the developmental transition of becoming an adult. Although TT has high semantic clarity and consistency, it is limited in its *structural clarity* and *consistency*. The relationships between concepts are not explained and can be confusing. In the model, the relationships are linear and bi-directional, but not all concepts are linked to each other. It was also found that there is considerable overlap between the properties of transition and transition conditions. For example, engagement and transition time span are properties of transition experiences; however, in the interviews, they were often endorsed as facilitators or inhibitors. In order for nurses to effectively intervene during transitions, they need to have a better understanding of the relationships between concepts.

*Simplicity* refers to the number of concepts and relationships present within a theory (Chinn & Kramer, 2015). Because TT contains many concepts and relationships, it can be judged as a fairly complex theory. This can be expected, as transition itself is a complex phenomenon that likely could not be described by a simple theory. Those who wish to utilize TT should be prepared to meet this challenge by considering interrelated dimensions of transitions rather than focusing on concepts in isolation. We considered the complexity of TT to be a strength, as it forced us to examine transition holistically.

*Generality* is defined in terms of a theory’s scope and purpose, with a general theory being applicable to a wide range of populations and/or situations (Chinn & Kramer, 2015). While the purpose of TT is meant to be broad, Meleis et al. (2000) also admit that the theory may not capture the complexities and uniqueness of diverse types of transitions. We believe that TT is limited in its generalizability to children and youth because it lacks a developmental lens. For example, *fluid integrative identities* may not be a realistic outcome indicator for children who are still discovering their self-identity. Even for older adolescents and emerging adults, identity conflict is a normal stage of development; thus, it is reasonable to believe that healthy transitions can be achieved without the formation of a fluid identity. By incorporating a developmental lens, TT would be more generalizable to younger populations.

*Accessibility* refers to the extent to which a theory’s concepts and relationships can be observed or experienced in real-world situations, with increasing complexity often leading to increased *accessibility* (Chinn & Kramer, 2015). Since TT is quite complex, it is also highly accessible for applications to practice and research. In the interviews, all concepts were present and resonated strongly as common themes. It was also found that many health and service providers are already delivering care that reflects the interventions outlined in Meleis’ (2015) intervention framework (clarifying roles, competencies, and meanings; identifying milestones; mobilizing support; and debriefing). This validates TT’s empiric accessibility and provides evidence that TT is capable of explaining some aspects of this particular transition. On the other hand, some of the relationships were not accurately depicted in this specific transition. For example, while the model implies that personal conditions and nursing therapeutics can affect the nature of transitions, in this situation, both nurses and families had little control over the types and patterns of transitions occurring. However, this may not be true for all transitions; thus, the theory’s ambiguous relationships should be made more explicit when considering the nuances specific to each transition.

The *importance* of a theory can be judged by its apparent and/or proven clinical significance and relevance to nursing. This criterion is highly subjective and will reflect the theory user’s values and beliefs about what is important to nursing (Chinn & Kramer, 2015). Because nurses frequently care for clients during different types of transitions, it can be argued that TT is highly important for nursing. People who are undergoing transitions are often at risk for poor health due to the need to adjust to significant changes. This is especially relevant for YMC, as transitions often result in the loss of relationships, trust, and connections to health and social services (Davies et al., 2011; Schultz, 2013), leading to further exacerbation of barriers to accessing care. Because the transition to adult services is a particularly salient issue for YMC, we believe that TT is an important theory for nurses working with this population.

A limitation of applying middle-range theory to practice is the need to translate the theory’s abstract ideas into something more concrete and actionable; the purpose for which the theory is applied may be more practical than the original purpose of the theory. While the original purpose of TT is to explain individuals’ experiences with transition, the aim of the project was to understand current practices and gaps and was not limited to families’ experiences. If we were to stay true to the original purpose of TT, then we might only interview family members to elicit perspectives from those who have direct experience of the transition. While understanding the experiences of families is instrumental to informing practice changes, their experiences alone are not enough to inform subsequent interventions. In conducting a needs assessment, it was important to understand the perspectives of as many stakeholders as possible, in order to appreciate the current practice landscape. The authors went beyond the original purpose of TT (understanding transition experiences) to achieve a more practical goal of understanding gaps and current practices, based on transition experiences. The authors could not change the practice issue to fit the theory; instead, the authors learned that using middle-range theories in practice requires flexibility and adaptation to address more concrete, practice-based issues.

Furthermore, while TT explained some aspects of the transition to adult services, there are several important elements of transition that are not captured by the original theory. To address these missing elements, the authors suggest incorporating additional concepts when considering this specific population and transition. First, as previously mentioned, family caregivers are heavily involved in care and decision-making for YMC. Often, this level of involvement is maintained throughout the transition process and continues into adulthood. Therefore, family conditions need to be considered, in addition to personal conditions.

Second, it is important to have a developmental lens when working with younger populations, which is not reflected in the original theory. *Nursing therapeutics* and *patterns of response* should be tailored to the youth’s developmental stage. However, research and testing are needed to determine what developmentally appropriate indicators related to transition should be.

Third, system issues were often endorsed as the biggest inhibitors to a healthy transition. Examples of system issues include waitlists that result in gaps in funding, general lack of options for individuals with both a developmental and physical disability or higher medical complexity, and geographical barriers to accessing care and services. Thus, the most influential way for nurses to promote healthy transitions may be through advocating for system-level changes (funding innovative solutions, breaking down silos, and involving youth and families in program development). Advocacy is an important concept in transitions and should be the joint responsibility of families, providers, communities, and societies.

Lastly, although TT is a nursing theory, it is relevant outside of the nursing discipline. In reality, nurses are only one part of an extensive network of individuals and services that support YMC and their families during transition. Additionally, it was found that providers from a variety of disciplines (medicine and social work), as well as lay persons, were delivering the *nursing* therapeutics described by Meleis’ (2015) intervention framework. These interventions are not unique to nursing, and we believe that changing *nursing therapeutics* to *therapeutic interventions* will increase TT’s generality.

This decision to remove *nursing* from the theory may be controversial and raises the questions, “Is this still a nursing theory?” and “What implications does this have for the future direction of the nursing discipline?” It is our belief that the theory would still be a nursing theory, as it still reflects nursing’s relational practice and foundational values. In a *Nursology.net* blog post, Peggy Chinn (2019) proposed that the defining features of a nursing theory are its focus on the two core elements of the discipline: “knowledge of the human health experience, and knowledge of nursing actions leading to health and well-becoming” (para. 4). Even after removing *nursing*, TT still focuses on the human health experience of transition, and *therapeutic interventions* are actions that can be taken by nurses, multidisciplinary providers, or lay persons that lead to health and well-becoming. Furthermore, in the current sphere of interdisciplinary health care practice, it may be valuable to have nursing theories that are accessible to a broader audience. This shift can help to increase awareness among our nonnursing colleagues of the unique contribution of nursing knowledge to the health sciences or, in the words of Liehr and Smith (2017), “bring middle range theory to the interdisciplinary table” (p. 61).

## Conclusion

In summary, this article provided an overview of TT and described and evaluated its application to a practice issue. Overall, TT was easy to apply and proved to be a useful framework for understanding the transition to adult services for YMC and their families. Although our overall experience with applying TT was positive, we discovered several limitations in applying the theory to our specific population and situation. Our use of the theory led to novel insights about highly important aspects of this particular transition that were missing from the original theory. Despite these limitations, we believe that TT allowed us to examine our practice issue more holistically than we would have in the absence of a theoretical basis. We support the use of theory to guide practice, but we recognize that theory users may need to tailor middle-range theories to serve more practical purposes and to address the many diverse practice issues that nurses encounter. In order for theory-based practice to occur, nurses need to be flexible in their use of theory and adept at critically evaluating the applicability of theory to their specific practice needs.

## Supplemental Material

sj-docx-1-nsq-10.1177_08943184211010454 – Supplemental material for Transitioning to Adult Services for Youth With Medical Complexity: A Practice Issue Viewed Through the Lens of Transitions Theory Click here for additional data file. Supplemental material, sj-docx-1-nsq-10.1177_08943184211010454 for Transitioning to Adult Services for Youth With Medical Complexity: A Practice Issue Viewed Through the Lens of Transitions Theory by Lin Li and Patricia H. Strachan in Nursing Science Quarterly

sj-pdf-2-nsq-10.1177_08943184211010454 – Supplemental material for Transitioning to Adult Services for Youth With Medical Complexity: A Practice Issue Viewed Through the Lens of Transitions Theory Click here for additional data file. Supplemental material, sj-pdf-2-nsq-10.1177_08943184211010454 for Transitioning to Adult Services for Youth With Medical Complexity: A Practice Issue Viewed Through the Lens of Transitions Theory by Lin Li and Patricia H. Strachan in Nursing Science Quarterly
