# Supplementary figures and images for: Transitioning to Adult Services for Youth With Medical Complexity: A Practice Issue Viewed Through the Lens of Transitions Theory
Source: Nurs Sci Q. 2021 Jul 2;34(3):301–8. doi: 10.1177/08943184211010454 (PMC8255503; doi:10.1177/08943184211010454)

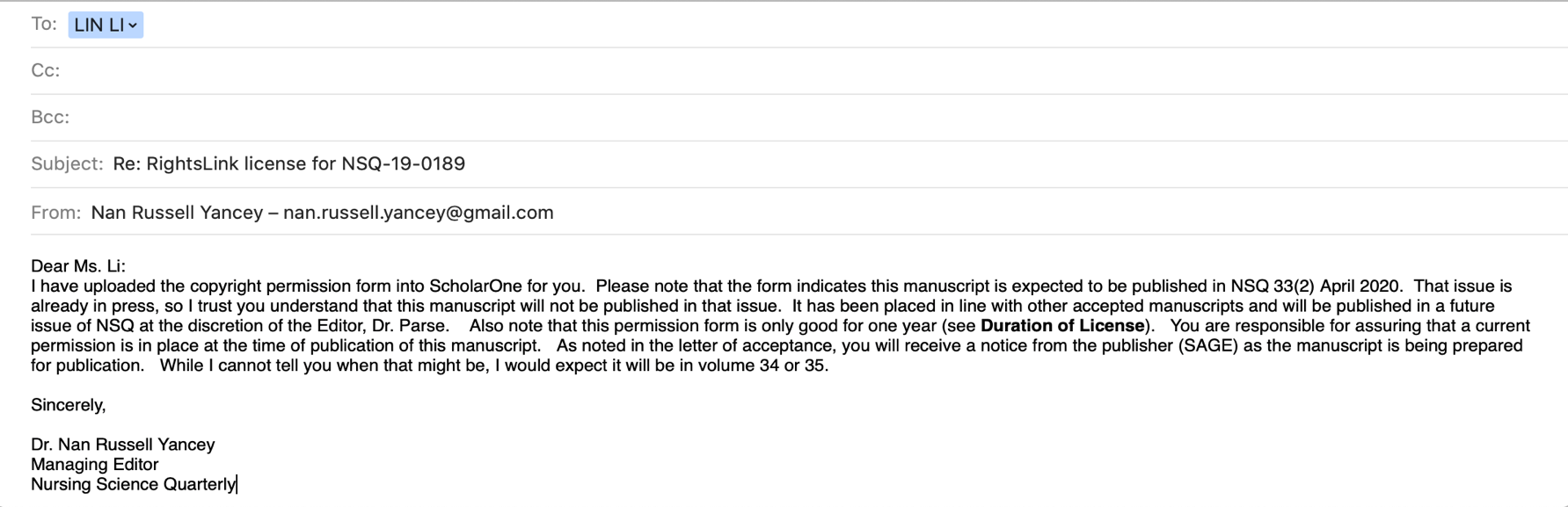

Supplement: sj-docx-1-nsq-10.1177_08943184211010454 – Supplemental material for Transitioning to Adult Services for Youth With Medical Complexity: A Practice Issue Viewed Through the Lens of Transitions Theory [file sj-docx-1-nsq-10.1177_08943184211010454.docx]
