# Supplementary material for: Transitioning to Adult Services for Youth With Medical Complexity: A Practice Issue Viewed Through the Lens of Transitions Theory
Source: Nurs Sci Q. 2021 Jul 2;34(3):301–8. doi: 10.1177/08943184211010454 (PMC8255503; doi:10.1177/08943184211010454)
Supplement: sj-pdf-2-nsq-10.1177_08943184211010454 – Supplemental material for Transitioning to Adult Services for Youth With Medical Complexity: A Practice Issue Viewed Through the Lens of Transitions Theory [file sj-pdf-2-nsq-10.1177_08943184211010454.pdf]

WOLTERS KLUWER HEALTH, INC. LICENSE TERMS AND CONDITIONS

Sep 27, 2019

This Agreement between Ms. Lin Li ("You") and Wolters Kluwer Health, Inc. ("Wolters Kluwer Health, Inc.") consists of your license details and the terms and conditions provided by Wolters Kluwer Health, Inc. and Copyright Clearance Center.

License Number	4677351162377
License date	Sep 27, 2019
Licensed Content Publisher	Wolters Kluwer Health, Inc.
Licensed Content Publication	Advances in Nursing Science
Licensed Content Title	Experiencing Transitions: An Emerging Middle-Range Theory.
Licensed Content Author	Meleis, Afaf; Ibrahim PhD, FAAN; Sawyer, Linda; PhD, RN; Im, Eun-Ok; PhD, RN; Hilfinger Messias, DeAnne; PhD, RN; Schumacher, Karen; PhD, RN
Licensed Content Date	Sep 1, 2000
Licensed Content Volume	23
Licensed Content Issue	1
Type of Use	Journal/Magazine
Requestor type	STM Signatory Publisher
STM publisher name	SAGE Publications
Format	Print and electronic
Portion	Figures/table/illustration
Number of figures/tables/illustrations	1
Figures/tables/illustrations used	Figure 1. Transitions: a middle-range theory
Author of this Wolters Kluwer article	No
Will you be translating?	No
Title of the content	Transitioning to Adult Services for Children and Youth with Medical Complexity: A Practice Issue Viewed Through the Lens of Transitions Theory
Publication the new content is in	Nursing Science Quarterly
Publisher of your content	SAGE Publications
Author of the content	Li, L. & Strachan, P. H.
Expected publication date	Jan 2020

Estimated size of content (pages)	15
Requestor Location	Ms. Lin Li<br>2 Giffin Rd<br><br><br>Dundas, ON L9H6S1<br>Canada<br>Attn: Ms. Lin Li
Total	0.00 CAD

Terms and Conditions

Wolters Kluwer Health Inc. Terms and Conditions

1. **Duration of License:** Permission is granted for a one time use only. Rights herein do not apply to future reproductions, editions, revisions, or other derivative works. This permission shall be effective as of the date of execution by the parties for the maximum period of 12 months and should be renewed after the term expires.
    i. When content is to be republished in a book or journal the validity of this agreement should be the life of the book edition or journal issue.
    ii. When content is licensed for use on a website, internet, intranet, or any publicly accessible site (not including a journal or book), you agree to remove the material from such site after 12 months, or request to renew your permission license
2. **Credit Line:** A credit line must be prominently placed and include: For book content: the author(s), title of book, edition, copyright holder, year of publication; For journal content: the author(s), titles of article, title of journal, volume number, issue number, inclusive pages and website URL to the journal page; If a journal is published by a learned society the credit line must include the details of that society.
3. **Warranties:** The requestor warrants that the material shall not be used in any manner which may be considered derogatory to the title, content, authors of the material, or to Wolters Kluwer Health, Inc.
4. **Indemnity:** You hereby indemnify and hold harmless Wolters Kluwer Health, Inc. and its respective officers, directors, employees and agents, from and against any and all claims, costs, proceeding or demands arising out of your unauthorized use of the Licensed Material
5. **Geographical Scope:** Permission granted is non-exclusive and is valid throughout the world in the English language and the languages specified in the license.
6. **Copy of Content:** Wolters Kluwer Health, Inc. cannot supply the requestor with the original artwork, high-resolution images, electronic files or a clean copy of content.
7. **Validity:** Permission is valid if the borrowed material is original to a Wolters Kluwer Health, Inc. imprint (J.B Lippincott, Lippincott-Raven Publishers, Williams & Wilkins, Lea & Febiger, Harwal, Rapid Science, Little Brown & Company, Harper & Row Medical, American Journal of Nursing Co, and Urban & Schwarzenberg - English Language, Raven Press, Paul Hoeber, Springhouse, Ovid), and the Anatomical Chart Company
8. **Third Party Material:** This permission does not apply to content that is credited to publications other than Wolters Kluwer Health, Inc. or its Societies. For images credited to non-Wolters Kluwer Health, Inc. books or journals, you must obtain permission from the source referenced in the figure or table legend or credit line before making any use of the image(s), table(s) or other content.
9. **Adaptations:** Adaptations are protected by copyright. For images that have been adapted, permission must be sought from the rightsholder of the original material and the rightsholder of the adapted material.
10. **Modifications:** Wolters Kluwer Health, Inc. material is not permitted to be modified or adapted without written approval from Wolters Kluwer Health, Inc. with the exception of text size or color. The adaptation should be credited as follows: Adapted with permission

from Wolters Kluwer Health, Inc.: [the author(s), title of book, edition, copyright holder, year of publication] or [the author(s), titles of article, title of journal, volume number, issue number, inclusive pages and website URL to the journal page].

11. **Full Text Articles:** Republication of full articles in English is prohibited.
12. **Branding and Marketing:** No drug name, trade name, drug logo, or trade logo can be included on the same page as material borrowed from *Diseases of the Colon & Rectum, Plastic Reconstructive Surgery, Obstetrics & Gynecology (The Green Journal), Critical Care Medicine, Pediatric Critical Care Medicine, the American Heart Association publications and the American Academy of Neurology publications.*
13. **Open Access:** Unless you are publishing content under the same Creative Commons license, the following statement must be added when reprinting material in Open Access journals: "The Creative Commons license does not apply to this content. Use of the material in any format is prohibited without written permission from the publisher, Wolters Kluwer Health, Inc. Please contact permissions@lww.com for further information."
14. **Translations:** The following disclaimer must appear on all translated copies: Wolters Kluwer Health, Inc. and its Societies take no responsibility for the accuracy of the translation from the published English original and are not liable for any errors which may occur.
15. **Published Ahead of Print (PAP):** Articles in the PAP stage of publication can be cited using the online publication date and the unique DOI number.
    i. Disclaimer: Articles appearing in the PAP section have been peer-reviewed and accepted for publication in the relevant journal and posted online before print publication. Articles appearing as PAP may contain statements, opinions, and information that have errors in facts, figures, or interpretation. Any final changes in manuscripts will be made at the time of print publication and will be reflected in the final electronic version of the issue. Accordingly, Wolters Kluwer Health, Inc., the editors, authors and their respective employees are not responsible or liable for the use of any such inaccurate or misleading data, opinion or information contained in the articles in this section.
16. **Termination of Contract:** Wolters Kluwer Health, Inc. must be notified within 90 days of the original license date if you opt not to use the requested material.
17. **Waived Permission Fee:** Permission fees that have been waived are not subject to future waivers, including similar requests or renewing a license.
18. **Contingent on payment:** You may exercise these rights licensed immediately upon issuance of the license, however until full payment is received either by the publisher or our authorized vendor, this license is not valid. If full payment is not received on a timely basis, then any license preliminarily granted shall be deemed automatically revoked and shall be void as if never granted. Further, in the event that you breach any of these terms and conditions or any of Wolters Kluwer Health, Inc.’s other billing and payment terms and conditions, the license is automatically revoked and shall be void as if never granted. Use of materials as described in a revoked license, as well as any use of the materials beyond the scope of an unrevoked license, may constitute copyright infringement and publisher reserves the right to take any and all action to protect its copyright in the materials.
19. **STM Signatories Only:** Any permission granted for a particular edition will apply to subsequent editions and for editions in other languages, provided such editions are for the work as a whole in situ and do not involve the separate exploitation of the permitted illustrations or excerpts. Please view: STM Permissions Guidelines
20. **Warranties and Obligations:** LICENSOR further represents and warrants that, to the best of its knowledge and belief, LICENSEE’s contemplated use of the Content as represented to LICENSOR does not infringe any valid rights to any third party.
21. **Breach:** If LICENSEE fails to comply with any provisions of this agreement, LICENSOR may serve written notice of breach of LICENSEE and, unless such breach is fully cured within fifteen (15) days from the receipt of notice by LICENSEE, LICENSOR may thereupon, at its option, serve notice of cancellation on LICENSEE, whereupon this Agreement shall immediately terminate.
22. **Assignment:** License conveyed hereunder by the LICENSOR shall not be assigned or granted in any manner conveyed to any third party by the LICENSEE without the consent in

writing to the LICENSOR.

23. **Governing Law:** The laws of The State of New York shall govern interpretation of this Agreement and all rights and liabilities arising hereunder.
24. **Unlawful:** If any provision of this Agreement shall be found unlawful or otherwise legally unenforceable, all other conditions and provisions of this Agreement shall remain in full force and effect.

For Copyright Clearance Center / RightsLink Only:

1. **Service Description for Content Services:** Subject to these terms of use, any terms set forth on the particular order, and payment of the applicable fee, you may make the following uses of the ordered materials:
   i. **Content Rental:** You may access and view a single electronic copy of the materials ordered for the time period designated at the time the order is placed. Access to the materials will be provided through a dedicated content viewer or other portal, and access will be discontinued upon expiration of the designated time period. An order for Content Rental does not include any rights to print, download, save, create additional copies, to distribute or to reuse in any way the full text or parts of the materials.
   ii. **Content Purchase:** You may access and download a single electronic copy of the materials ordered. Copies will be provided by email or by such other means as publisher may make available from time to time. An order for Content Purchase does not include any rights to create additional copies or to distribute copies of the materials

Other Terms and Conditions:
v1.18

Questions? customercare@copyright.com or +1-855-239-3415 (toll free in the US) or +1-978-646-2777.
